# Correction: Marchant et al. Ankle Somatosensation and Lower-Limb Neuromuscular Function on a Lunar Gravity Analogue. *Brain Sci.* 2025, *15*, 443

**DOI:** 10.3390/brainsci15101098

**Published:** 2025-10-13

**Authors:** Ashleigh Marchant, Nick Ball, Jeremy Witchalls, Sarah B. Wallwork, Gordon Waddington

**Affiliations:** 1 Research Institute for Sport and Exercise, University of Canberra, Canberra, ACT 2617, Australia; nick.ball@canberra.edu.au (N.B.); gordon.waddington@canberra.edu.au (G.W.); 2 IIMPACT in Health, University of South Australia, Adelaide, SA 5001, Australia

## Missing Conflicts of Interest

This update pertains to the article previously published by Marchant et al. [1]. In the original publication, there were missing sentences in the “Conflicts of Interest” section.

The correct sentences appear below:

**Conflicts of Interest:** The author Gordon Waddington is a founder and shareholder in Prism Neuro Pty Ltd., an Australian perceptual neuroscience equipment company. The author declares that the research was conducted in the absence of any commercial or financial relationships that could be construed as a potential conflict of interest.

The authors state that the scientific conclusions are unaffected. This correction was approved by the Academic Editor. The original publication has also been updated.
